# Health-Related Physical Fitness Benefits in Sedentary Women Employees after an Exercise Intervention with Zumba Fitness^®^

**DOI:** 10.3390/ijerph17082632

**Published:** 2020-04-11

**Authors:** Yaira Barranco-Ruiz, Emilio Villa-González

**Affiliations:** Department of Physical and Sports Education, PROFITH “PROmoting FITness and Health through Physical Activity” Research Group, Sport and Health University Research Institute (iMUDS), Faculty of Education and Sport Sciences, University of Granada, Melilla 52071, Spain; evilla@ugr.es

**Keywords:** physical activity, sedentarism, group-based fitness class, physical fitness, health

## Abstract

*Background*: The protective properties of high physical fitness levels on health are manifest independently of age, sex, fatness, smoking, alcohol consumption, and other clinical factors. However, women are less active than men, which contributes to low physical fitness and a high risk of CVD. Thus, the objective of this study is to analyze the effect of two exercise interventions on health-related physical fitness in sedentary employed females. *Methods*: A total of 98 physically inactive adult women were randomly assigned to 3 study groups: the control group (CG) = 31, the endurance training group based on Zumba Fitness^®^ classes (ET; *n* = 39), and the concurrent training group that had an extra muscle-strengthening workout after the Zumba Fitness^®^ class (CnT; *n* = 28). Body composition (BMI, waist circumference), motor fitness (one-leg stand test), musculoskeletal fitness (shoulder–neck mobility, handgrip strength test, jump and reach test, and dynamic sit-up test), and cardiorespiratory fitness (2-km test and estimated VO_2_ max) were assessed with the ALPHA-Fit test battery for adults. *Results*: Both interventions showed a significant improvement in the majority of health-related fitness and body composition variables/test (waist circumference: ET = −2.275 ± 0.95, *p* = 0.02, CnT = −2.550 ± 1.13, *p =* 0.03; one-stand leg test: ET = 13.532 ± 2.65, *p* < 0.001; CnT = 11.656 ± 2.88, *p* < 0.001; shoulder–neck mobility: ET = 1.757 ± 0.44, *p* < 0.001, CnT = 2.123 ± 0.47, *p* < 0.001; handgrip test: 0.274 ± 0.08, *p* < 0.001, CnT = 0.352 ± 0.08, *p* < 0.001; dynamic sit-up: ET = 1.769 ± 0.45, *p* < 0.001, CnT = −1.836 ± 0.49, *p* < 0.001; 2-km test time: −1.280 ± 0.25, *p* < 0.001, CnT = −1.808 ± 0.28, *p* < 0.001; estimated VO_2_ max: ET = 1.306 ± 0.13, *p* < 0.001, CnT = 1.191 ± 0.15, *p* < 0.001). There were no significant differences after the intervention between the two exercise intervention groups. *Conclusions*: Exercise interventions based on Zumba fitness^®^ or Zumba fitness^®^ combined with an extra muscle-strengthening workout based on bodyweight training exercises are effective strategies to improve the health-related physical fitness in sedentary women employees.

## 1. Introduction

The World Health Organization (WHO) acknowledges physical inactivity as a global public health burden, representing the fourth-leading risk factor for global death, above high-blood pressure, smoking, and high-blood glycemia [1]. Evidence of the positive effects of physical activity in the prevention of many chronic diseases has been chiefly demonstrated [2], with long-term improvements in physical fitness, overall health benefits, as well as protection from premature death [3]. Additionally, the protective properties of high physical fitness levels on health are manifest independently of age, sex, fatness, smoking, alcohol consumption, and other clinical factors, and are directly associated with high levels of physical activity [4]. The recommendations of the American College of Sports Medicine (ACSM) to decrease/maintain a healthy body weight and enhance cardiovascular fitness include 75 to 150 min of weekly exercise [5]. ACSM also recommends adding at least two days of full-body muscle-strengthening activities [5]. However, reports on physical activity trends in Europe show a plateau in sport and physical activity participation in the past few years, especially in women [6]. Recently, another report published the worldwide trends of insufficient physical activity from 2001 to 2016, where Latin America and the Caribbean women reached the highest physical inactivity levels in 2016 (43.7%) [7]. In addition, nowadays, job occupations enclose a high prevalence of sedentary tasks, which contributes to the increase inactive time [8] and, thus, a low-calorie expenditure. Therefore, several strategies to reduce overall sedentary time and improve body composition and health through physical activity programs have been aimed in the community of employees (especially in women) over the last few years [9,10,11]. Previous studies have recognized several barriers associated with inactivity behavior in women at midlife, such as low income, time constraints, or education [6,12,13,14].

Dance, on the other hand, is the second most popular leisure-time physical activity after walking among women across all ages (25 to 75 years plus) [15]. It is also an activity recommended in the recent Global Action Plan On Physical Activity 2018–2030 established by WHO [16]. In addition, substantial benefits of dancing activities on physiologic, endocrine, cognitive, and psychological levels have previously been disclosed [17,18,19,20,21,22]. Thus, it is indispensable to promote strategies based on novel and attractive activities, such as dancing, for increasing physical activity and assuring adherence and self-efficacy in physical activity patterns in women. In this sense, Zumba Fitness^®^ is a dance-based fitness class that originated in Colombia in the 1990s and is now extremely popular worldwide (www.zumbafitness.com). Scientific studies about the potential benefits of Zumba Fitness^®^ in women have revealed many positive effects of this type of workout on body composition and physical fitness [23,24,25], which is therefore considered a very successful program for encouraging physical activity levels among sedentary women. A recent review of the literature [26], highlighted that even Zumba Fitness^®^ could be considered as a type of aerobic training, improving the aerobic and cardiovascular outcomes, since it had a promising small-but-positive effect on body composition, muscular strength, balance, and quality of life. However, results differed between studies due to heterogeneity among the investigations, workout intensities, and intervention periods. Therefore, more research is needed to clarify whether the effect of the Zumba Fitness^®^ program can achieve improvements beyond aerobic and cardiovascular outcomes.

Finally, to our knowledge, there are few studies that evaluate the effect of different intervention programs based on Zumba Fitness^®^ on health-related markers such as physical fitness. Among all these types of interventions, we will focus on two: (i) an intervention where the Zumba Fitness^®^ modality is applied in isolation, and thus, where it is mainly endurance training (ET), or (ii) an intervention where an extra muscle-strengthening part with bodyweight exercises is included after the Zumba Fitness^®^ part, and therefore, it is considered a concurrent training (CnT), where endurance and strength are combined in the same training session. We considered it interesting to compare the effect of these two types of interventions (ET and CnT) since this type of muscle-strengthening training with the bodyweight is included in several fitness centers in the last minutes of a group-based choreographic fitness class, but its effect on physical fitness is still unknowing. We hypothesize that both exercise interventions (i.e., ET and CnT) would be linked to changes in different health-related physical fitness dimensions at the end of the intervention compared to the control group. Thus, the objective of this study is to analyze and compare the effect of two exercise interventions based on mainly Zumba Fitness^®^ classes (with or without additional muscle-strengthening training with their own body weight) on health-related physical fitness in physically inactive employed females.

## 2. Methods

### 2.1. Study Design

A randomized control trial with a 3 × 2 statistical design was carried out for this study. It included two experimental groups based on different exercise interventions and one control group (no exercise intervention) and two moments of evaluation: (1) at baseline and( 2) after 16 weeks of the intervention period.

### 2.2. Participants

Adult women (25 to 50 years) employees at the university were invited through email to this study. Inclusion criteria were apparently healthy women (without a diagnosis of any disease or pathology), with more than 6 h of sedentary tasks in their habitual working day, inactive women (less than 150 min of moderate-to-vigorous physical activity per week), no experience in group-based fitness programs, not having practiced any physical or sports activity in more than one year, and with availability and compromise to attend the majority of the exercise sessions (70%) during the intervention period (16 weeks). The exclusion criterion was the diagnosis or suffering of serious diseases such as cancer, stroke, heart attack, and serious musculoskeletal illness. After the email invitation, the first screening was performed in two initial and informative study sessions. From 150 interested women who attended these sessions, only 120 agreed to participate in the study, and, finally, 98 women accomplished the inclusion criteria and attended the initial evaluations. After these evaluations, participants were randomly assigned to study groups. Randomization was blinded to the research team. The methodology followed for randomization was as follows: one person outside the research team randomly pulled out ballots one by one from a big central box and distributed them in three other boxes (one for each study group). In the big central box, there was one ballot per participant with her corresponding code. Researchers did not know the code number assigned to the participants or what intervention represented each box. The ballot was always deposited in the left-to-right direction. The study was conducted in accordance with the Declaration of Helsinki, and the protocol was approved by the Ethics Committee on Research of the National University of Chimborazo (Ecuador) (Ref: 29-CI-2014-10-17-22). Each participant gave her informed consent before the beginning of the study.

### 2.3. Procedures and Interventions

All participants allocated to their corresponding study group performed 16 weeks of intervention. This study presented two experimental groups with two different exercise interventions. One exercise intervention was mainly based on endurance training (ET group), and the other exercise intervention was a concurrent training (CnT group), including an extra-strength workout. The ET exercise intervention group performed a group-based Zumba Fitness^®^ classes for one hour. Zumba Fitness ^®^ classes were led by a certified instructor (ZIN) and mainly followed the choreographies from Zumba Fitness^®^ ZIN DVD number 48 and 50. Zumba fitness^®^ classes (approx. 60 min in total) consists of 3 parts: (i) a warm up of 10 min with choreographies principally based on mobility, short displacements, and dynamic stretching (music from 125 to 135 bpm); (ii) a main part (40–45 min) based on the combination of different choreographies with Latin rhythms (merengue, salsa, reggaeton, music from 140 to 180 bpm); and a final cool down (5–10 min) with choreographies based on soft Latin rhythms (bachata or salsa, music from 120–135 bpm) combined with breathing and stretching movements. All participants performed the Zumba Fitness^®^ sessions at the same time. The CnT group continued with 20 extra minutes of muscular strength training based on a choreographed workout with bodyweight exercises that focused on functional movements of main muscle groups, such as lower limbs, chest, upper limbs, abdomen, and lower back. Participants executed these movements following the rhythm of five music tracks of approximately 4 min each one (one music track by each muscle group/2 or 3 exercises per muscle group during each music track). Both exercise interventions were carried out at 6:00 p.m., three days per week in the sports facilities of the university with musical and choreographed support. The intensity of the sessions was assessed by the 0 to 10 rating perceived exertion (RPE) Borg scale [27]. The certified Zumba fitness^®^ instructor regularly informed us about the intensity ranges and changes, following the mentioned scale, which allowed the control of the intensity levels within moderate-to-vigorous intensity ranges (from 6–8 in the 0 to 10 RPE Borg Scale), ensuring that subjects were not exposed to strenuous efforts. In order to analyze the adherence to the exercise interventions, attendance was registered every session with the participants’ signature before and after them, as well as the average of the intensity according to the RPE Borg scale at the end of each session. The control group continued with its habitual lifestyle without exercise intervention. Furthermore, the three study groups received two healthy nutrition habit seminars (45 min each) by a professional nutritionist (weeks 1 and 8 during the intervention period). During these seminars, participants explored their nutritional habits, and they received a talk and an informative triptych with healthy recommendations. Baseline and post-intervention body composition evaluations were performed at the university medical center in the morning in fasting conditions and not having exercised for at least the previous 48 h. Physical fitness assessments were carried out the same day in the afternoon at the sports facilities of the university.

Health-related fitness was assessed with the ALPHA-FIT battery for adults from 18 to 69 years old [28]. This battery presented several dimensions of physical fitness and tests. The dimensions were (a) body composition, (b) motor fitness, (c) musculoskeletal fitness, and (d) cardiorespiratory fitness. For this study, the following tests and variables were used to analyze the health-related fitness.

*(a)* Body composition

Body mass index (BMI) is calculated as the weight (kg) divided by the square of height (m). Nutritional status from BMI was presented based on the Consensus of the Spanish Society for Obesity Research (Spanish Society for the Study of Obesity): Underweight: BMI ≤ 18.5; Normal weight: BMI from 18.5 to 24.9; Overweight: BMI from 25 to 29.9; Obesity I: BMI from 30 to 34.9; Obesity II: BMI from 35 to 39.9; Morbid Obese: BMI ≥ 40; Extreme Obesity: BMI ≥ 50) [29]. Waist circumference was assessed according to the National Heart, Lung, and Blood Institute (NHLBI) and the WHO protocols [30,31] considering cardiovascular risk when the waist circumference  was ≥80 cm, according to cut points for South Latin-American women [32].

*(b)* Motor fitness

The motor fitness was evaluated by the one-leg stand test whose purpose is to evaluate the postural control and balance on one foot. The evaluator first demonstrated the correct performance to the participants and had each participant practice to demonstrate they were able to perform the test. As a practice, the participant stood on each leg and chose the best one for the test. For the correct execution of this test, the participant should stand on one leg with their free leg resting against the inner side of their supporting leg with the free-heel in the middle of the supporting knee joint. Arms had to be relaxed and loose next to the trunk and may be used to help to keep balance only when necessary. The participant stared at one fixed point and could not use any side/surface as support. Two trials were performed if the result of the first trial was less than 60 s. The evaluator started timing when the participant reached the correct test position. Timing stops when the client loses the balance or at the maximal duration of the test (60 s). This test has three fitness categories: Low Fit (from 0 to 26 s), Mid fit (from 30 to 59 s), and High fit (60 s).

*(c)* Musculoskeletal fitness

The musculoskeletal dimension has several tests. Shoulder–neck mobility, handgrip, jump-and-reach, and dynamic sit-up were the tests used in this study.

The shoulder–neck mobility test estimates the restrictions in functional mobility of the shoulder-neck region. The participants stand with their back and buttocks against the wall and feet placed at ½ foot length from the wall. The participants have to raise their arms above their head and try to touch the wall with the backs of their hands. Scores for the right and left sides were taken separately. 5 points = no restriction of range of motion (touch the wall with the full back of their hands), 3 points = moderate restriction of range of motion (touch the wall with the partial back of their hands), and 1 point = severe restriction of range of motion (not being able to touch the wall). The final score defines the fitness categories and it is obtained from the sum of both sides. There are five fitness categories (Lowest fit = 0–2 points, Low fit = 3–4 points, Mid fit = 5–6 points, High fit = 7–8 points, Highest fit = 9–10 points).

The handgrip test measures the static grip strength with a hand dynamometer (Smedley III digital, USA) and is an indicator of the strength in upper limbs and overall strength [28]. The participant is standing with the dynamometer in the hand of her choice and with the arm extended and slightly separated from the trunk. The grip of the dynamometer is adjusted to the size of the hand to bring the second joint of the forefinger approximately to a right angle. The participants have to squeeze the dynamometer firmly and gradually, building quickly up to maximal force for 4 s. The evaluator registers the score and the participant rapidly changes hand and repeats the same process. The participant performs four attempts without stops. The final score is the mean resulting from the four attempts. Lifting the arm or strange movements with the rest of the body are not allowed. The handgrip strength was expressed as a relative measure (newtons) according to the bodyweight of each participant. There are five fitness categories (Lowest fit, Low fit, Mid fit, High fit, Highest fit) according to the final score depending on age and gender [28].

The jump and reach test evaluates the leg extensor power and strength of the lower limbs. The participant has to do a vertical jump as high as possible with her dominant upper extremity, raising up straight, and touching a board, as high as possible, with her fingers. The vertical difference between the “standing height” and the “jumping height” is measured in centimeters with a tape measure. There are four fitness categories to evaluate the score of this test (Low fit, Low-to-Mid fit, Mid fit, High fit) depending on age and gender [28].

The dynamic sit-up test has the purpose of evaluating the dynamic strength of the abdominal and hip flexor muscles. Lying supine on a mat with knees flexed aligned with the ankles, the participant must flex his trunk in three different ways. In the first five attempts, participants have to reach their mid patella with the fingertips keeping the arms straight. In the second sit-ups, participants have to reach their mid patella with both elbows, keeping their arms crossed on the chest. In the last five sit-ups, participants must touch their mid patella with both elbows keeping their arms flexed and their hands touching the back of their earlobes. This test has three fitness categories: Low Fit (if participant only gets 5 first sit-up), Mid fit (correctly perform the five more = 10 sit-ups), and High fit (perfectly perform 15 sit-ups).

*(d)* Cardiorespiratory fitness

The cardiorespiratory fitness was measured through the 2-km walking test. The objective of this test is to predict maximal oxygen uptake (VO_2_max) and to measure the ability of brisk walking. The participant must walk as fast as possible on a flat surface along 2 km. Heart rate at rest before the test and heart rate and time instantly after crossing the finish line were registered. Heart rate was measured with a pulsometer (model Polar m400, Polar Electro company, Kempele, Finland) and a heart rate sensor (model H7, by Polar Electro company, Kempele, Finland). Running is not allowed. The estimated maximal oxygen uptake (VO_2_max) was calculated from the following equation: (1) Multiply and add the values: [walking time (min) × 8.5] + [walking time (s) × 0.14] + [heart rate (beats/min) × 0.32] + [BMI (kg/m2) × 1.1] = sum; (2) Subtract from the sum: sum - [age (years) × 0.4] = difference; (3) Subtract the calculated difference: 304 − difference = Fitness Index. This test has four fitness categories (Low fit, Low-to-Mid fit, Mid fit, High fit) depending on age/gender [28] and extracted from the total time in the test.

### 2.4. Statistical Analysis

Data are expressed as mean (M) and standard deviation (SD), or mean differences (MD) and standard error of the mean (SEM), or marginal mean difference and standard error of the difference (SE diff) for quantitative variables according to the statistical test. Descriptive variables are presented as frequency and percentage. The distribution of the study variables was analyzed with the Kolmogorov–Smirnov test. All analyses were performed with the IBM SPSS package 25.0 (IBM Corp, Armonk, NY, USA). A descriptive and comparative analysis was performed for the anthropometric characteristics of the sample at baseline. The one-way ANOVA test was used for analyzing the mean comparisons according to the study groups for quantitative variables when the variables had a normal distribution or with the Kruskal–Wallis test when they had a non-normal distribution. The chi-square test was used for these types of comparisons in categorical variables. Per protocol, statistical analyses were used. A mixed factorial ANOVA test was carried out to assess the effect of the intervention on health-related physical fitness variables (quantitative variables), the main effects of the study factors (study groups and measure moments), as well as the possible interactions between the study factors. The Bonferroni test was used for pair comparisons, and all analyses were adjusted for baseline value and age. The partial eta-squared (η^2^) was interpreted as “small” effect (0.01); “small-to-medium” effect (0.01–0.10); “medium-to-large” (0.10–0.25). Within-group effect size was calculated using “Cohen’s d” and interpreted as follows: small effect (0.10); small-to-medium (0.10–0.25); and medium-to-large effect (≥0.25). Changes in categorical variables were analyzed with the McNemar test. For all analyses, *p*-value was established at <0.05.

## 3. Results

### 3.1. Characteristics of the Sample at Baseline

The characteristics of the sample at baseline are presented in Table 1. The mean age of the sample was 38.69 ± 7.29 years old (minimum 25 years, maximum 50 years, mode 42 years old) without statistical differences between the study groups. Adulthood, according to chronological age (early adulthood = less than 40 years old, and late adulthood = more than 40 years old), was balanced between the study groups without statistical differences. The mean for the bodyweight of the sample was 66.06 ± 12.22 kg, and there were no differences between study groups. The same occurred for height (meters), waist circumference (cm), and BMI (kg/m^2^), where there were no significant differences between study groups. The values for the mean of the sample in these variables were as follows: 1.59 ± 0.11 m, 84.23 ± 10.48 cm, and 26.04 ± 3.89 kg/m^2^. Normal weight and overweight were the most prevalent categories among study groups; however, there were no statistical differences in the distribution of the nutritional status categories between the study groups. In general, more than half of the study sample presented cardiovascular risk (CVR) according to the value of waist circumference (62.80%), with no statistical differences between study groups. Regarding physical fitness dimensions, there were statistical differences between study groups only for the shoulder–neck mobility test, handgrip strength test, and estimated VO_2_ max. For that reason, analyses were adjusted for the baseline value of each participant in each variable in the baseline-post-intervention comparisons.

### 3.2. Health-Related Physical Fitness after the Intervention and According to Study Groups

A total of 76 participants successfully completed the baseline and post-intervention assessments (control group: *n* = 22, ET: *n* = 31, CnT: *n* = 23), with a dropout of 22.4% (control group: *n* = 11, ET: *n* = 2 and CnT: *n* = 9). The main reasons for declination of participation were changes in work schedule and appearance of illness, accident (non-related to intervention), or special conditions such as pregnancy.

**Figure 1:**
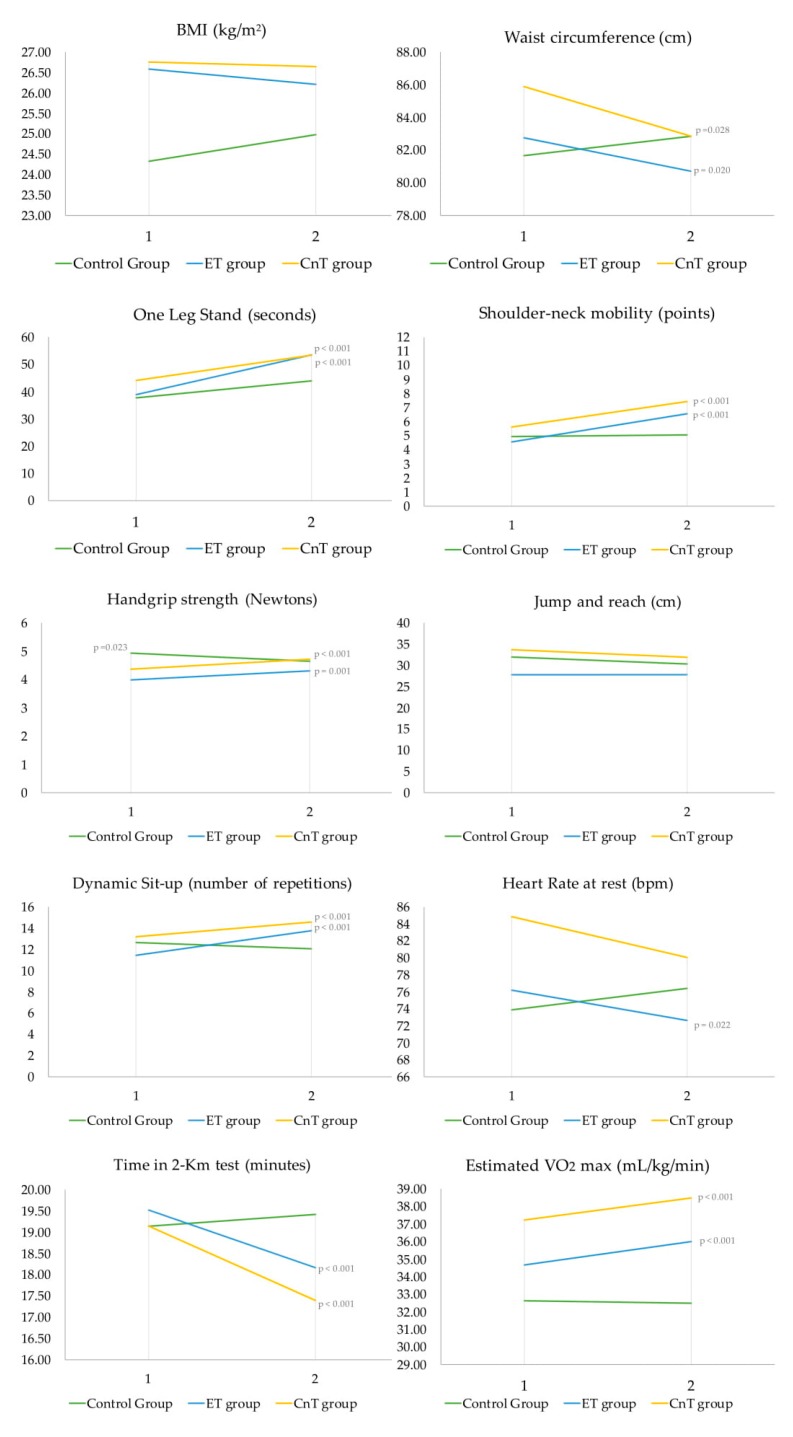
Health-related physical fitness variables at baseline (1) and post-intervention (2), according to participants who completed baseline and post-intervention assessments (*n* = 76) by study groups (control group, *n* = 22; ET group, *n* = 31, CnT group, *n* = 23). ET group = endurance training group; CnT = concurrent training group.

The means of the health-related physical fitness components at baseline and post-intervention, as well as the interactions between study factors are presented in Figure 1. Some significant interactions were observed. The control group showed a statistical interaction with both exercise intervention groups in the shoulder–neck mobility test values (F (1, 70) = 4.438, *p* = 0.016, η^2^*p* = 0.133), handgrip strength values (F (1, 70) = 11.470, *p* < 0.001, η^2^*p* = 0.287), the dynamic sit-up test values (F (1, 70) = 5.565, *p* = 0.006, η^2^*p* = 0.161), the time in 2-km test (F (1, 70) = 13.166, *p* < 0.001, η^2^*p* = 0.316),and in the estimated VO_2_ max (F (1, 70) = 21,629, *p* < 0.001, η^2^*p* = 0.436).

The differences between baseline and post-intervention in the marginal means within the study groups and the effect size are presented in Table 2. Regarding pre-post changes within study groups, no significant changes were observed for the control group except in handgrip strength, where participants showed a significant decrease compared with baseline values (*p* = 0.023). Both exercise intervention groups (ET and CnT) displayed significant post-intervention improvements in the one-leg stand test, shoulder–neck mobility, handgrip strength, dynamic sit-up test, time in the 2-km test, and estimated VO_2_ max (Table 2). No statistically significant changes were observed for any of the study groups in the jump and reach test. In the case of the heart rate at rest, only the ET group showed significant improvement compared with baseline values. Comparing the study groups after the intervention (post-test), the control group statistically differed only from the CnT group in shoulder-neck mobility (MD = 1.007 ± 0.36 points; *p* = 0.19). In the case of the handgrip strength test, the control group showed statistical differences in post-intervention compared with both exercise intervention groups (control group versus ET group: MD = 0.497 ± 0.130, *p* = 0.001; control group versus CnT group: MD = 0.575 ± 0.124, *p* < 0.001). The same was observed with the sit-up test (control group versus ET group: MD = −2.109 ± 0.719, *p* = 0.014; control group versus CnT group: MD = 2.176 ± 0.732, *p* = 0.013), the time in 2-km test, and estimated VO_2_ max (control group versus ET group: MD = 1.515 ± 0.396, *p* = 0.001; control group versus CnT group: MD = 2.043 ± 0.409, *p* < 0.001).

Finally, the average of RPE during sessions was 7.5 (0.74) for both exercise groups (ET group = 7.26 (0.73) and CnT group = 7.81 (0.64). The % of attendance to exercise interventions was 77.7 ± 6.7 for the ET group and 78.2 ± 8.1 for the CnT group.

**Figure 2:**
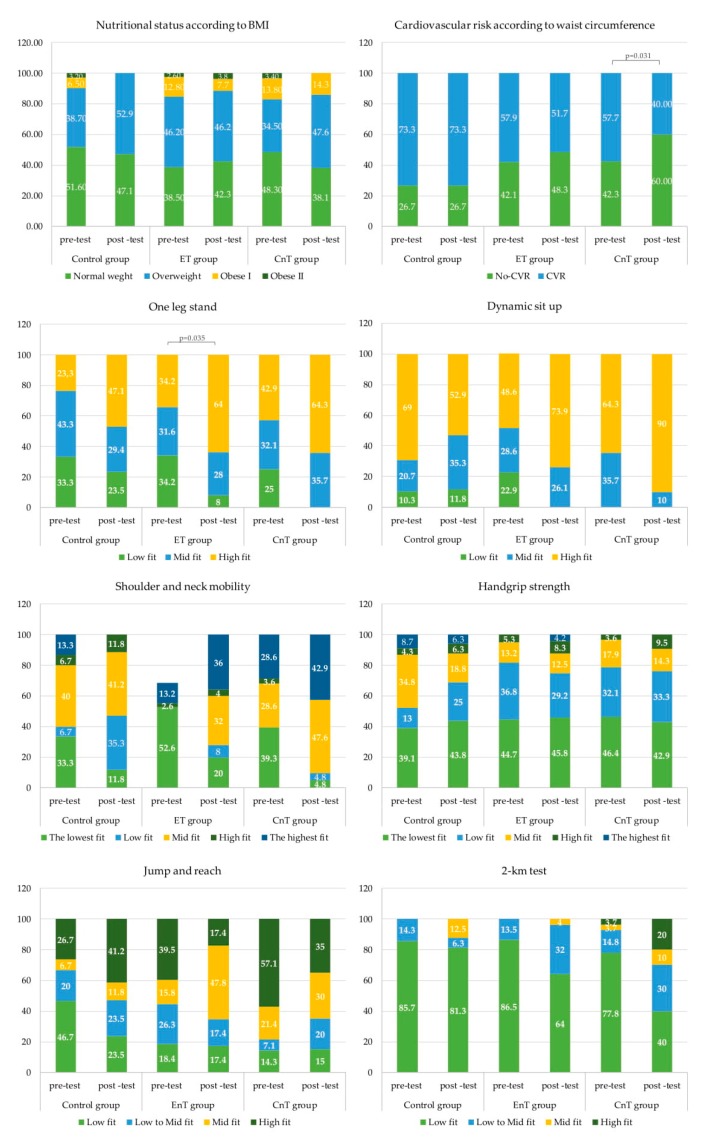
Changes in health-related physical fitness categories according to study groups. ET group = endurance training group; CnT = concurrent training group.

According to the health-related fitness categories, a significant improvement was shown by the the CnT group in the CVR based on the waist circumference value. The ET group also showed a significant improvement in fitness categories of the one-leg stand test. No statistical changes regarding nutritional status categories and the rest of physical fitness tests were observed according to the study groups (Figure 2).

## 4. Discussion

The main findings of this study were that both exercise intervention groups (Zumba Fitness^®^ and the concurrent training including an extra-strength workout) statistically improved their waist circumference, one-leg stand test, shoulder–neck mobility, handgrip strength, dynamic sit up, time in the 2-km test and the estimated VO_2_ max scores, whereas the control group displayed no significant changes after the intervention period. Additionally, significant interactions were observed between the control group and both exercise intervention groups for shoulder and neck mobility, handgrip strength, dynamic sit up, time in the 2-km test, and estimated VO_2_ max, since both exercise groups improved and the control group did not improve or get worse.

Regarding changes in the health related-physical fitness post-intervention in the present study, both exercise intervention groups experienced significant changes in several physical fitness dimensions, whereas the control group showed no significant changes in most of them. The majority of the studies based on Zumba Fitness^®^ intervention programs have shown solid improvement in aerobic capacity in comparison with the other physical fitness outcomes.

In our study, both exercise intervention groups showed statistical improvements in the cardiorespiratory fitness test scores, i.e., time in the 2-km test as well as in the estimated VO_2_ max after the 16 weeks of intervention. Additionally, only the ET group presented statistical improvement after intervention in HR at rest. Also, significant interactions were observed between the control group and both exercise intervention groups for the time in the 2-km test and the estimated VO_2_ max. Improvements in cardiorespiratory fitness were not homogeneous across previous studies, from approximately 1% [23] to a noteworthy 19% after 12 weeks of training [24]. Differences between studies could be due to different reasons, such as the exercise intensity or adequate training progression during the interventions. Also, intermediate assessments, especially in longer procedures, permit us to modify the aerobic intensity and then to produce more relevant aerobic increases. In the present study, Zumba Fitness^®^ classes were led by a certified instructor (ZIN) following a progressive training based on the original structure of Zumba Fitness^®^, and this fact could have generated greater control and organization within the classes, possibly related to significant changes in cardiovascular outcomes. Moreover, in our study, the intensity was assessed by the RPE Borg scale at the end of each class with a consistent progression, ensuring at least moderate-to-vigorous intensity on average in both exercise groups. Most studies that analyze changes in physical fitness components through a Zumba Fitness^®^ intervention did not control or register/inform about the intensity training variable [33,34,35,36]. Only two studies included in the previously mentioned review monitored HR, but none quantified the exercise intensity, and there were no descriptions of any training progression. Likewise, most of the studies directly and indirectly measured aerobic capacity, whereas only a few studies measured it with field-based tests [26], as in the present study. Thus, the results describe the potential positive effects of Zumba fitness^®^ on aerobic capacity; however, although the findings were promising, further analyses including objective intensity control are recommended.

Concerning musculoskeletal fitness, both exercise intervention groups showed statistical differences in the handgrip strength test and the dynamic sit-up *test* after the 16-weeks intervention. Also, significant interactions were observed between the control group and both exercise intervention groups for the same variables. Most of the studies that evaluated muscular strength (e.g., upper or lower limb) did not show significant improvements after a Zumba Fitness^®^ intervention [26]. Only one study reported an increase in the lower limb strength [37]; however, the improvement was not high (15%). Specifically, in our study, both the handgrip strength test and the dynamic sit-up test scores improved after the period of intervention in both exercise groups. However, there were no differences between the ET group and the CnT group in pre-post changes for theses variables. In this sense, it is important to highlight the reason why, in the present study, two different exercise interventions were carried out. Since there is extensive literature focused on the benefits of strength training on physical fitness in adult women [38], we wanted to analyze the extra effect of strength training, beyond the benefits of the Zumba Fitness^®^ intervention. In addition, the recent review based on the health benefits of Zumba Fitness^®^ [26] included interventions with muscle strengthening exercises after the main part of Zumba Fitness^®^ at the same session (although the official structure of Zumba Fitness^®^ class does not contemplate it). Therefore, it is interesting to analyze the effect of this additional strength training, usually without external loads (bodyweight training), to isolate the individual effects of an official Zumba Fitness^®^ class on health-related variables. Commonly, Zumba Fitness^®^ appeared to be efficient as an activity to improve cardiorespiratory fitness. The nonexistence of a form of overload (lightweight to moderate dumbbells, weight discs, bar with weight, ankle weight or elastic bands) during the class would be doubtlessly the main reason explaining the no increase in musculoskeletal fitness in the majority of the studies. However, in the current study, both intervention programs (with and without strength training) generated significant improvements in the strength of the upper limbs, as well as the strength-resistance of the core muscle trunk after the intervention period. We could hypothesize that since the sample of sedentary women presented low levels of physical fitness at baseline (below the European average in both cases, i.e., handgrip and sit-up test) [28], it could generate rapid and palpable improvements in strength in just 16 weeks of intervention in both groups. This fact can also be explained because endurance training can improve strength fitness and vice versa, and both capacities can be increased indirectly, especially in sedentary subjects [39]. In addition, a 16-week period of intervention using Zumba Fitness^®^ probably influenced other physical variables beyond aerobic capacity, due to the fact that this type of training is very global (i.e., a multi-joint and full-body muscle workout). As the whole body is trained at a moderate-to-vigorous intensity and includes jumps, displacements, core exercises, and choreographies with effusive arm movements, among others, the improvement of muscle strength could be possible from a starting low level. However, as mentioned earlier, the generated impact by the CnT intervention (Zumba fitness^®^ + extra muscle strengthening with bodyweight exercises) may not be enough strength stimulus to generate differences compared with the ET (only Zumba Fitness^®^ classes). So, future interventions should include external load elements (beyond body weight training), such as dumbbells or bars to increase the total training load, if the objective is to significantly influence musculoskeletal variables.

With respect to shoulder-neck mobility, to our knowledge, there are very few studies based on Zumba Fitness^®^ interventions evaluating this functional capacity in the musculoskeletal dimension. However, it is an interesting measure since greater mobility improvements could be expected due to the wide range of motion of the shoulder–neck, waist, and hip joints with the movements involved in the choreographies of Zumba Fitness^®^ classes. In the present study, the control group statistically differed only from the CnT group in the shoulder–neck mobility after the intervention, which reached the greater differences from baseline, although without statistical differences compared with the ET intervention (Zumba fitness^®^ isolated). In this sense, different studies in adults have reported improvements in this physical fitness variable after a strength training program, but mostly they were focused on a physical rehabilitation program [40,41]. As the effect of the strength training program was not evaluated separately, it is not possible to know which program produced the improvements in this variable. Moreover, although the improvements could be attributed to the program with strength training, the Zumba Fitness^®^ intervention could also generate improvements in the range of motion, particularly in shoulders. For instance, a previous study carried out in twenty-eight overweight/obese women described improvements in the hamstrings and low back ranges of motion after the 16-week Zumba Fitness^®^ intervention [37], but not in shoulder or neck mobility. Further, as in the present study, Zumba Fitness^®^ encourages participants to include flexibility movements at the end of each training session, during the cool-down phase in order to improve this functional capacity; however, most of the studies do not usually report the specific information about movements or exercises for this phase.

Finally, the current study evaluated *motor fitness*, including *the one-leg stand test*. There were significant differences after the intervention in the seconds to perform the test in both exercise groups (both *p* < 0.001) with a medium-to-large effect size. In addition, according to the health-related physical fitness categories, a significant improvement was shown in the ET group for the one-leg stand test (*p* = 0.035). To our knowledge, only one study measured balance after a Zumba Fitness^®^ intervention with similar results. In this study, the authors evaluated the dynamic balance ability in different body positions with the Star Excursion Balance test and improvements were observed in all balance test examinations [25]. With these results and with our findings, it can be speculated that dynamic activity based on nonstop and speedy movements characterizing Zumba Fitness^®^ workout could have determined an increase in dynamic balance control. However, the comparison between studies must be carried out with caution, since variables such as age (female college students in the Donath et al. (2013) study vs. sedentary adults females in our study) or training attendance (100% in the study of Donath et al. (2013) vs. 78% in our study) could clearly influence the results.

Most investigations based on Zumba Fitness^®^ described a light-to-moderate reduction in body weight ranging from 1% to 3.6% of the total body weight mass [26]. Similarly, most of the analyzed studies in that review detected a decrease in BMI. In the current study, only waist circumference (cm) displayed significant changes, but with a low effect size. With regard to waist circumference, two previous studies showed a moderate reduction [37,42], implying a reduced risk factor. In fact, high waist circumference is associated with all-cause mortality in healthy middle-aged women according to BMI ranges between 25 and 34.9 kg/m^2^ [43]. Additionally, a significant improvement was shown by the CnT group in the CVR based on the waist circumference value, according to the health-related fitness categories, but there were no changes regarding nutritional status categories after interventions in the study groups. Interestingly, the largest improvements in both body weight and BMI in the previously mentioned studies were identified in the overweight and obese samples, similarly to our study, where obese/overweight participants showed greater losses (BMI = from −0.17 to 0.65 kg/m^2^, and BW = from 0.25 to 0.98 kg) (data not shown). Differences in anthropometric parameter changes could be due to the variability of workout durations or frequencies of the Zumba Fitness^®^ classes.

Some limitations must be recognized in the present study. First, the selection process was by convenience, although a randomized intervention assignment was done. Second, in terms of intensity control, we could have used a more objective measure, such as a pulsometer; however, we have used the RPE Borg Scale as a valid method. As a strength, this is a study with a strong statistical analysis adjusted for baseline values and age. Additionally, although there was a dropout of participants, it did not exceed the 25%, which is considered a fatal defect in the quality analysis of intervention studies for the promotion of health through the Effective Public Health Practice Project EPHPP [44]. Also, the adherence of the participants was considerably high (78%). In addition, this study analyzes the effects of two intervention fitness programs on health-related physical fitness, which have been little studied in sedentary women employees despite being widely practiced as a fitness activity trends.

## 5. Conclusions

In conclusion, a 16-week exercise intervention (3 times per week/1 h) based on Zumba Fitness^®^ (with or without an extra muscle strengthening workout) is an effective strategy to improve health-related physical fitness in sedentary women employees. An extra muscle strengthening workout, including bodyweight exercises, that addresses the additional ACSM component of physical activity recommendations, did not produce an extra effect on health-related physical fitness beyond the Zumba Fitness^®^ classes.

## Figures and Tables

**Table 1:** Characteristics of the sample at baseline.

	Overall Sample (*n* = 98)		Control Group (*n* = 33)		ET Group (*n* = 33)		CnT Group (*n* = 32)		Statistical Differences between Groups
	M	SD	M	SD	M	SD	M	SD	*p*-Value
Age	38.69	7.29	38.06	7.11	38.06	7.11	38.47	6.67	0.734
*Adulthood*									
Early adulthood (18 to 40 years old) *	52	52.90	20	60.60	18	46.20	16	53.30	0.470
Late adulthood (>40 years old) *	46	47.10	13	39.40	21	53.80	14	46.70	
*Anthropometric parameters*	98								
BW (kg)	66.06	12.22	64.25	12.35	64.25	12.35	68.85	13.65	0.320
Height (meters)	1.59	0.11	1.59	0.14	1.59	0.14	1.62	0.11	0.338
Waist circumference (cm)	84.23	10.48	85.90	10.35	85.90	10.35	83.55	9.93	0.573
BMI (Kg/m^2^)	26.04	3.89	25.44	3.46	25.44	3.46	26.26	3.76	0.583
*Nutritional status*									
Normal weight *	44	45.50	16	51.60	15	38.50	14	48.30	0.890
Overweight *	40	40.40	12	38.70	18	46.20	10	34.50	
Obese I *	11	11.10	2	6.50	5	12.80	4	13.80	
Obese III *	3	3.00	1	3.20	1	2.60	1	3.40	
*Cardiovascular risk according to waist circumference*									
No CVR *	35	37.20	8	26.70	16	42.10	11	42.30	0.350
CVR *	59	62.80	22	73.30	22	57.90	15	57.70	
*Physical Fitness dimensions*									
* Motor fitness *									
One leg stand (seconds)	40.31	19.42	36.59	18.87	40.41	18.89	43.90	20.64	0.300
*Musculoskeletal Fitness*									
Shoulder-neck mobility (points)	4.97	3.03	4.97	2.82	4.47	2.92	5.64	3.36	**<0.001**
Handgrip strength (newtons)	4.33	1.02	4.84	0.91	3.93	1.03	4.38	0.88	**0.002**
Jump-and-reach (cm)	30.65	7.75	30.81	9.05	28.37	5.04	33.57	8.51	0.110
Dynamic sit-up (number of sit-up)	12.43	3.79	12.90	3.98	11.40	4.18	13.25	2.76	0.150
*Cardiorespiratory Fitness*									
Heart rate in rest (bpm)	78.26	13.27	76.00	11.19	77.03	13.83	82.29	14.01	0.750
Total time in 2-km test (min)	29.46	16.26	26.69	17.40	30.30	16.36	31.30	15.03	0.460
Estimated VO_2_ max (mL/kg/min)	34.94	5.38	32.63	5.90	34.67	3.94	37.23	5.74	**0.030**

ET group = endurance training group; CT = concurrent training group; BW = bodyweight; BMI = body mass index; CVR = cardiovascular risk. Data are expressed as mean (M) and standard deviation (SD) for quantitative variables, and frequency and percentage for qualitative variables (*) for an initial sample of 98 participants. Statistical differences between groups for quantitative variables by one-way ANOVA and by chi-squared for categorical variables, *p*-value < 0.005 in bold.

**Table 2:** Health-related physical fitness changes post-intervention within the study groups.

Health-Related Physical Fitness	Control Group (*n* = 22)					ET (*n* = 31)					CnT (*n* = 23)				
	Marginal Mean Diff (SE Diff)			*p*-Value	Effect Size	Marginal Mean Diff (SE diff)			*p*-Value	Effect Size	Marginal Mean Diff (SE Diff)			*p*-Value	Effect Size
***Body composition***															
BMI (kg/m^2^)	0.52	±	0.29	0.073	0.22	−0.34	±	0.23	0.100	0.01	−0.05	±	0.25	0.84	0.06
Waist circumference (cm)	0.97	±	1.30	0.458	0.17	−2.275 *	±	0.95	**0.020**	0.21	−2.550 *	±	1.13	**0.03**	0.10
*Motor fitness*															
One leg stand (seconds)	4.79	±	3.32	0.155	0.33	13.532 *	±	2.65	**0.000**	0.93	11.656 *	±	2.88	**0.000**	0.54
*Musculoskeletal Fitness*															
Shoulder-neck mobility (points)	0.09	±	0.53	0.867	0.06	1.757 *	±	0.44	**0.000**	0.65	2.123 *	±	0.47	**0.000**	0.60
Handgrip strength (newtons)	−0.223 *	±	0.10	**0.023**	0.33	0.274 *	±	0.08	**0.001**	0.46	0.352 *	±	0.08	**0.000**	0.41
Jump-and-reach (cm)	−1.50	±	1.00	0.140	0.17	0.440	±	0.85	0.605	0.00	1.35	±	0.91	0.143	0.19
Dynamic sit-up (number of sit-up)	−0.34	±	0.55	0.536	0.14	1.769 *	±	0.45	**0.000**	0.71	1.836 *	±	0.49	**0.000**	0.64
*Cardiorespiratory Fitness*															
Heart rate in rest (bpm)	0.41	±	2.25	0.855	0.25	−4.296 *	±	1.82	**0.022**	0.31	−2.08	±	2.09	0.324	0.33
Total time in 2-km test (min)	0.23	±	0.30	0.441	0.16	−1.280 *	±	0.25	**0.000**	1.20	−1.808 *	±	0.28	**0.000**	0.84
Estimated VO_2_ max (mL/kg/min)	−0.03	±	0.16	0.869	0.04	1.306 *	±	0.13	**0.000**	0.23	1.191 *	±	0.15	**0.000**	0.23

ET group = endurance training group; CnT = concurrent training group. Data are expressed as marginal mean differences and standard error differences. * Statistical differences *p* < 0.05 between baseline and post-intervention values of health-related fitness variables through factorial mixed ANOVA are presented in bold letters. Within-group effect size of the exercise interventions was calculated using Cohen’s d and interpreted as follows: small effect (0.10), small-to-medium (0.10–0.25), and medium-to-large effect (≥0.25).
